# 951. Implementation and Rapid Uptake of an Antimicrobial Stewardship Clinical Decision Support System Across 19 UPMC Hospitals

**DOI:** 10.1093/ofid/ofac492.794

**Published:** 2022-12-15

**Authors:** J Ryan Bariola, Tina Khadem, Caley Yakemowicz, Courtney Simonick, Erin K McCreary, Christina Andrzejewski, John W Mellors, Rima Abdel-Massih

**Affiliations:** UPMC, Pittsburgh, Pennsylvania; UPMC, Pittsburgh, Pennsylvania; Infectious Diseases Connect, pittsburgh, Pennsylvania; Infectious Diseases Connect, pittsburgh, Pennsylvania; University of Pittsburgh School of Medicine, Pittsburgh, Pennsylvania; Infectious Diseases Connect, pittsburgh, Pennsylvania; University of Pittsburgh School of Medicine, Pittsburgh, Pennsylvania; UPMC, Pittsburgh, Pennsylvania

## Abstract

**Background:**

UPMC Antimicrobial stewardship (AS) includes independently functioning AS programs and a central tele antimicrobial stewardship (TASP) (1 ID pharmacist and 1 ID physician) that supports smaller hospitals with few on-site AS resources. We describe implementation of an AS clinical decision support system (CDSS) at 19 UPMC hospitals (12-695 staffed beds). Figure 1 is an example of the CDSS.

**Figure 1:**
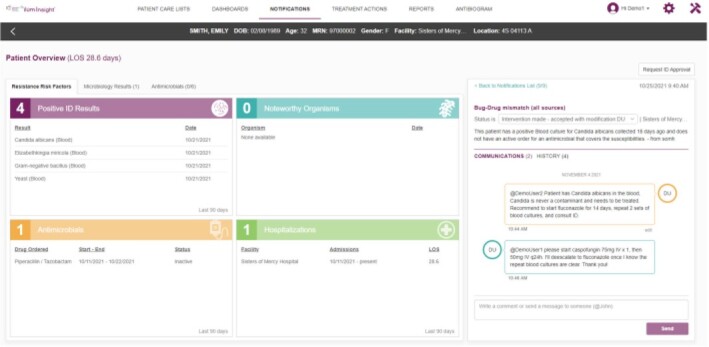
Representative Example of CDSS Patient Overview Page (not real patient data)

**Methods:**

The CDSS extracts clinical data to provide real-time alerts to AS teams. It also supports asynchronous communications between AS team members, including between a local AS and our TASP. CDSS utilization and intervention data from Oct 21 to Mar 22 are reported. Figure 2 shows hospital locations and the timeline for CDSS implementation. Table 1 shows implementation steps.

**Figure 2:**
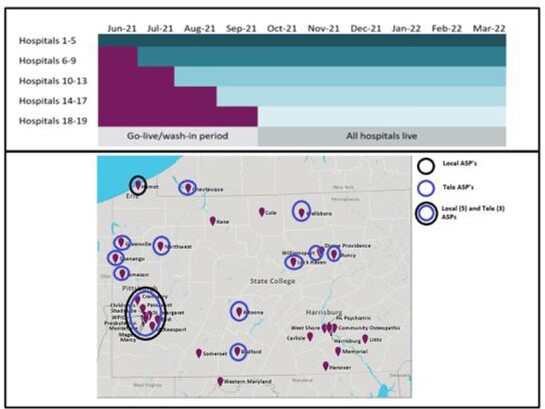
Implementation Timeline and Geographic Location of Hospitals

**Table 1:**
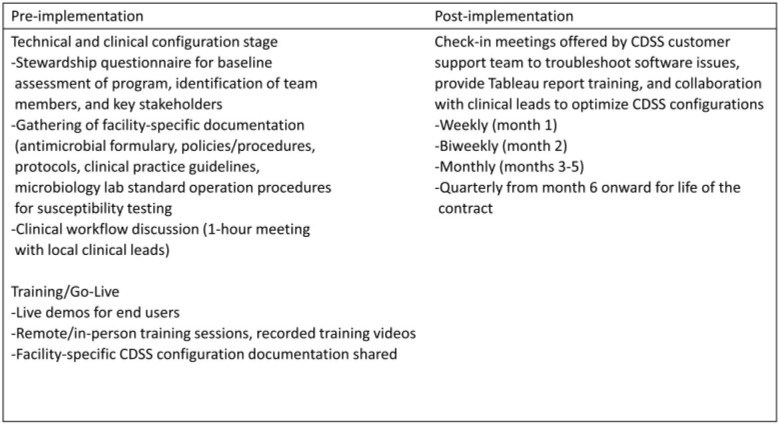
Implementation Steps

**Results:**

Table 2 summarizes category alerts provided by the CDSS. Category 1 alerts were independently reviewed by local pharmacists. Category 2 alerts were reviewed either by local pharmacists or central TASP team. Figure 3 shows high CDSS usage over time with numerous unique users and frequent logins. Most users were pharmacists. CDSS usage was steady and increased in the final month included here. Communications between users were frequent and increased 46%. On average, 7033 CDSS generated alerts per month (70-798 per hospital) were reviewed from Oct 21 to Mar22. Table 3 groups the alerts into successful, unsuccessful, or nonactionable interventions. 93% of actionable alerts (959 of 1029 per month) produced a successful outcome.

**Table 2:**
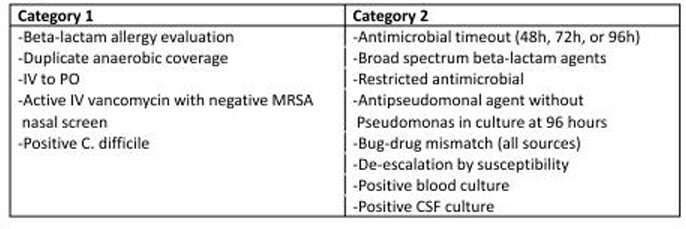
CDSS Alert Categories

**Figure 3:**
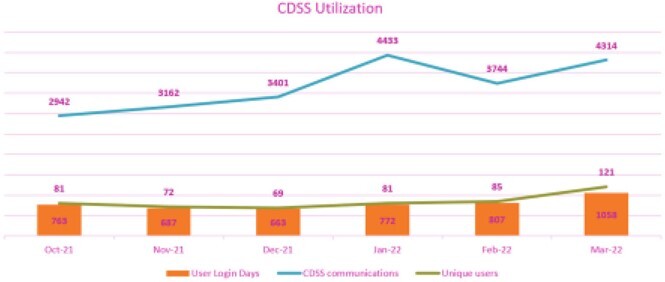
CDSS Utilization Summary

**Table 3:**
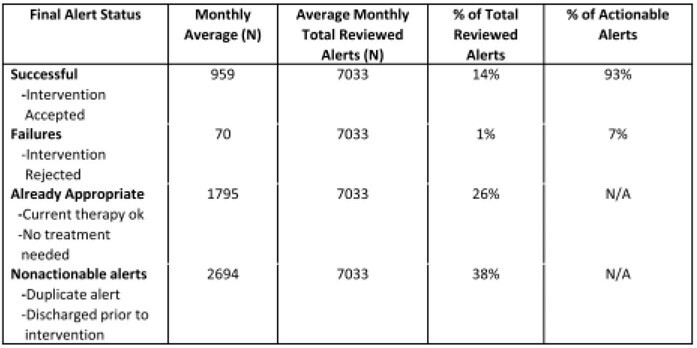
Summary and Alert Statuses and Intervention Outcomes

**Conclusion:**

After a rapid 5-month deployment across 19 hospitals this CDSS was successfully adopted as evidenced by sustained monthly use, increasing communications, and a high rate of accepted interventions on actionable alerts. A high rate of nonactionable alerts in the first 6 months was a limitation. Although this did not appear to limit CDSS use, sites implementing a CDSS should monitor for nonactionable alerts during early utilization and adjust as needed. CDSS has the potential to improve efficiency and consistency of AS within a health system. After a successful implementation, evaluation of impact on antimicrobial usage and clinical outcomes are important future metrics.

**Disclosures:**

**J Ryan Bariola, MD**, Infectious Disease Connect: Salary support|Merck: Grant/Research Support **Tina Khadem, PharmD**, Infectious Disease Connect: Salary support|Merck: Grant/Research Support **Caley Yakemowicz, n/a**, Infectious Disease Connect: Employee **Courtney Simonick, n/a**, Infectious Disease Connect: Stocks/Bonds **Erin K. McCreary, PharmD**, AbbVie: Advisor/Consultant|Cidara: Advisor/Consultant|Entasis: Advisor/Consultant|Ferring: Advisor/Consultant|Merck: Advisor/Consultant|Shionogi: Advisor/Consultant|Summit: Advisor/Consultant **Christina Andrzejewski, n/a**, Melinta Therapeutics: employee **John W. Mellors, MD**, Abound Bio: Multiple|Abound Bio: Ownership Interest|Gilead Sciences: Advisor/Consultant|Infectious Disease Connect: Advisor/Consultant|Infectious Disease Connect: Ownership Interest **Rima Abdel-Massih, MD**, Infectious Disease Connect: Co founder and Chief Medical Officer|Infectious Disease Connect: Ownership Interest.

